# Supplementary material for: Meta-optics redefines microdisplay: monolithic color LCoS without polarization dependency
Source: Nat Commun. 2025 Dec 8;16:10925. doi: 10.1038/s41467-025-66032-z (PMC12685945; doi:10.1038/s41467-025-66032-z)
Supplement: Supplementary file 1 — Supplementary Information [file 41467_2025_66032_MOESM1_ESM.pdf]

Supplementary Information for:

Meta-Optics Redefines Microdisplay: Monolithic Color LCoS without Polarization Dependency

Xiangnian Ou[1,#], Yueqiang Hu[1,2,3,#,*], Dian Yu[1], Shulin Liu[1], Shaozhen Lou[1], Zhiwen Shu[2], Wenzhi Wei[1], Man Liu[1], Jianxiong Li[4], Tianhai Chang[4], Na Liu[5*], and Huigao Duan[1,2*]

[1] National Research Center for High-Efficiency Grinding, College of Mechanical and Vehicle Engineering, Hunan University, Changsha 410082, P.R. China

[2] Greater Bay Area Institute for Innovation, Hunan University, Guangzhou 511300, P.R. China

[3] Advanced Manufacturing Laboratory of Micro-Nano Optical Devices, Shenzhen Research Institute, Hunan University, Shenzhen 518000, P.R. China

[4] Huawei Technologies Co., Ltd., Bantian, Longgang District, Shenzhen 518129, P.R. China

[5] 2nd Physics Institute, University of Stuttgart, Pfaffenwaldring 57, 70569 Stuttgart, Germany

[#]These authors contributed equally to this work.

[*]Corresponding authors. Email: huyq@hnu.edu.cn; na.liu@pi2.uni-stuttgart.de; duanhg@hnu.edu.cn

Section 1: Polarization-insensitive phase modulation

The polarization-insensitive property of the LC-covered columns can be explained in terms of the transmission matrix which can be expressed:

$$T = \begin{pmatrix} \mathrm{e}^{\mathrm{i}\varphi_{\mathrm{p1}}} & 0 \\ 0 & \mathrm{e}^{\mathrm{i}\varphi_{\mathrm{p2}}} \end{pmatrix} R(45°) \begin{pmatrix} \mathrm{e}^{\mathrm{i}\varphi_{\mathrm{m}}} & 0 \\ 0 & \mathrm{e}^{\mathrm{i}(\varphi_{\mathrm{m}}+\pi)} \end{pmatrix} R(-45°) \begin{pmatrix} \mathrm{e}^{\mathrm{i}\varphi_{\mathrm{p1}}} & 0 \\ 0 & \mathrm{e}^{\mathrm{i}\varphi_{\mathrm{p2}}} \end{pmatrix}$$
$$= \mathrm{e}^{\mathrm{i}(\varphi_{\mathrm{m}}+\varphi_{\mathrm{p1}}+\varphi_{\mathrm{p2}})} \begin{pmatrix} 0 & 1 \\ 1 & 0 \end{pmatrix}$$

where $R(\theta) = \begin{pmatrix} \cos\theta & -\sin\theta \\ \sin\theta & \cos\theta \end{pmatrix}$ is the rotation matrix. The electric field of the emitted light can be expressed as $\mathbf{E}_{\mathrm{o}} = T\mathbf{E}_{\mathrm{i}}$. $\mathbf{E}_{\mathrm{i}}$ denotes the electric field of the incident light. Unpolarized light can be considered a random combination of different types of polarized light, such as linear, circular, and elliptical polarization.

When linearly polarized light is incident:

$$\mathbf{E}_{\mathrm{i}} = \begin{pmatrix} \cos\alpha \\ \sin\alpha \end{pmatrix}, \mathbf{E}_{\mathrm{o}} = T\mathbf{E}_{\mathrm{i}} = \mathrm{e}^{\mathrm{i}(\varphi_{\mathrm{m}}+\varphi_{\mathrm{p1}}+\varphi_{\mathrm{p2}})} \begin{pmatrix} \sin\alpha \\ \cos\alpha \end{pmatrix}$$

When circularly polarized light is incident:

$$\mathbf{E}_{\mathrm{i}} = \begin{pmatrix} 1 \\ \pm i \end{pmatrix}, \mathbf{E}_{\mathrm{o}} = T\mathbf{E}_{\mathrm{i}} = \mathrm{e}^{\mathrm{i}(\varphi_{\mathrm{m}}+\varphi_{\mathrm{p1}}+\varphi_{\mathrm{p2}})} \begin{pmatrix} \pm i \\ 1 \end{pmatrix}$$

When elliptically polarized light is incident:

$$\mathbf{E}_{\mathrm{i}} = \begin{pmatrix} A\mathrm{e}^{\mathrm{i}\varphi_x} \\ B\mathrm{e}^{\mathrm{i}\varphi_y} \end{pmatrix}, \mathbf{E}_{\mathrm{o}} = T\mathbf{E}_{\mathrm{i}} = \mathrm{e}^{\mathrm{i}(\varphi_{\mathrm{m}}+\varphi_{\mathrm{p1}}+\varphi_{\mathrm{p2}})} \begin{pmatrix} B\mathrm{e}^{\mathrm{i}\varphi_y} \\ A\mathrm{e}^{\mathrm{i}\varphi_x} \end{pmatrix}$$

The matrix of the emitted light shows that the incident light with different polarizations is converted into light with orthogonal polarization after modulation and reflection by the device, accompanied by the same phase modulation independent of polarization, which is consistent with the previous analysis results.

Section 2: Metasurface design

The intensity of the off-axis reflection point generated by the super unit cell is modulated by subwavelength interference in the x-direction, depending on the overall phase difference $\Delta\varphi_x$. The phase difference $\Delta\varphi_x$ is the sum of the geometric phase difference $\Delta\varphi_\mathrm{g}$ of the nanorods and the propagation phase $\Delta\varphi_\mathrm{p}$ between the nanograting and the LC, as illustrated in Supplementary Fig. 1. When $\Delta\varphi_x = (2j\text{-}1)\pi$ $(j = 1, 2, 3 \cdots)$, complete destructive interference occurs, resulting in the reflected light deflected into the evanescent wave domain and exhibiting an "off" state. Conversely, as $\Delta\varphi_x$ deviates from π, the reflected light reappears, fully switching "on" when $\Delta\varphi_x = 2j\pi$.

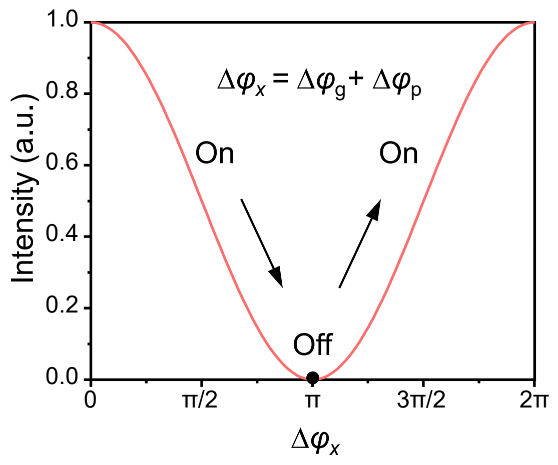

Supplementary Fig. 1. Calculated results of the intensity of the off-axis reflection point modulated by $\Delta\varphi_x$. The overall phase difference in the x-direction $\Delta\varphi_x = 2j\pi$ and $\Delta\varphi_x = (2j\text{-}1)\pi$ of the super unit cell corresponds to the "on" and "off" states of the optical switch, respectively.

When a plane wave from the z-direction is reflected after normal incidence onto the metasurface device, the reflected wave vector can be expressed as follows

$$\mathbf{k}_z = \sqrt{\mathbf{k}_0^2 - \mathbf{k}_x^2 - \mathbf{k}_y^2}$$

where k_0 is the wave vector of the plane wave, $\mathbf{k}_x=2\pi/P_x$, and $\mathbf{k}_y=2\pi/P_y$ are the wave vector provided by the metasurface along *x*- and *y*-directions, respectively. $2\pi/P_x$ and $2\pi/P_y$ are the phase gradient along the *x*- and *y*-directions, respectively. As illustrated in Supplementary Fig. 2, the circular boundary defined by $\left(\frac{\lambda}{P_x}\right)^2 + \left(\frac{\lambda}{P_y}\right)^2 = 1$ separates the two-dimensional spatial spectrum into a propagation wave domain and an evanescent wave domain. Within this framework, light is reflected when $\mathbf{k}_z > 0$ and deflected into the evanescent wave domain when $\mathbf{k}_z < 0$. Consequently， by meticulously designing the phase gradient along the *x*- and *y*-directions, the off-axis reflection light can be entirely deflected into the evanescent wave domain, thereby achieving the “off” state of the polarization-insensitive meta-LCoS device. This portion of the light is coupled as the SPP wave, with the corresponding energy absorbed by the metasurface.

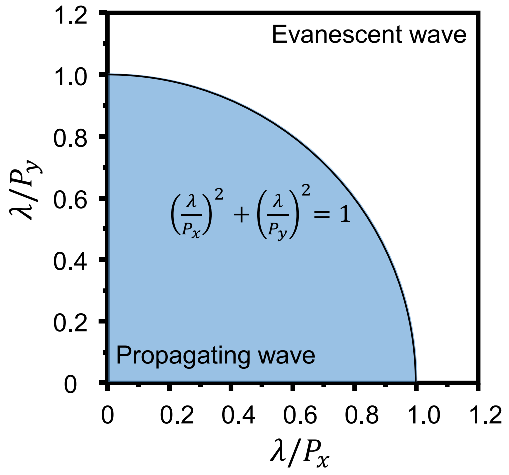

Supplementary Fig. 2. Two-dimensional spatial frequency spectrum. The blue region denotes the propagating wave, while the white region represents the evanescent wave.

Section 3: Comparative analysis of methodologies for realizing optical switch

Supplementary Fig. 3 illustrates the specific structure design of the super unit cell, along with the corresponding phase profiles and resulting display intensities. Three possible methodologies for realizing polarization-insensitive optical switch are considered and discussed. Supplementary Fig. 3a illustrates a straightforward methodology. The geometrical phase of the nanorods is employed in the *x* direction to construct the phase difference $\Delta\varphi_{\mathrm{g}} = \pi$. By meticulously regulating the height of the nanograting and selecting optimal LC, the propagation phase difference $\Delta\varphi_{\mathrm{p}} = (n_{\mathrm{LC}}\text{-}n_{\mathrm{G}})\mathbf{k}d$ between the nanograting and the LC is set to $\Delta\varphi_{\mathrm{p1}} = 0$ and $\Delta\varphi_{\mathrm{p2}} = \pi$. The phase gradient in the *x*-direction is utilized to generate off-axis reflection light, thereby achieving the "off" state. This precise geometric phase difference, introduced by the nanorod rotation, stabilizes the "off" state, enabling high contrast performance. The methodology is designed to be simple and easy to implement. However, the presence of zero-order light results in reduced contrast. Furthermore, to enhance contrast, a linear phase gradient ($\frac{\mathrm{d}\phi}{\mathrm{d}y} = \frac{2\pi}{4P}$, where *P* is the lattice period of single unit cell) is introduced along the *y*-direction to form an off-axis reflection point as the switching point that distinguish from zero-order diffraction point, as illustrated in Supplementary Fig. 3b. This methodology has the same configuration as Supplementary Fig. 3a, but achieves a higher contrast ratio. However, due to the phase gradient in the *x*-direction, a significant number of higher-order diffraction spots appear in the projection, which can substantially reduce the efficiency of the

meta-LCoS and impair the quality of the projected image. Finally, we propose the methodology illustrated in Supplementary Fig. 3c as a solution to the aforementioned problem. By optimizing the lattice period of the nanorods to be less than half of the wavelength of the incident light, the off-axis reflection light is deflected into the evanescent wave domain, thereby reducing higher-order diffraction. The specific design method is presented in the main text and Supplementary Information Section 2.

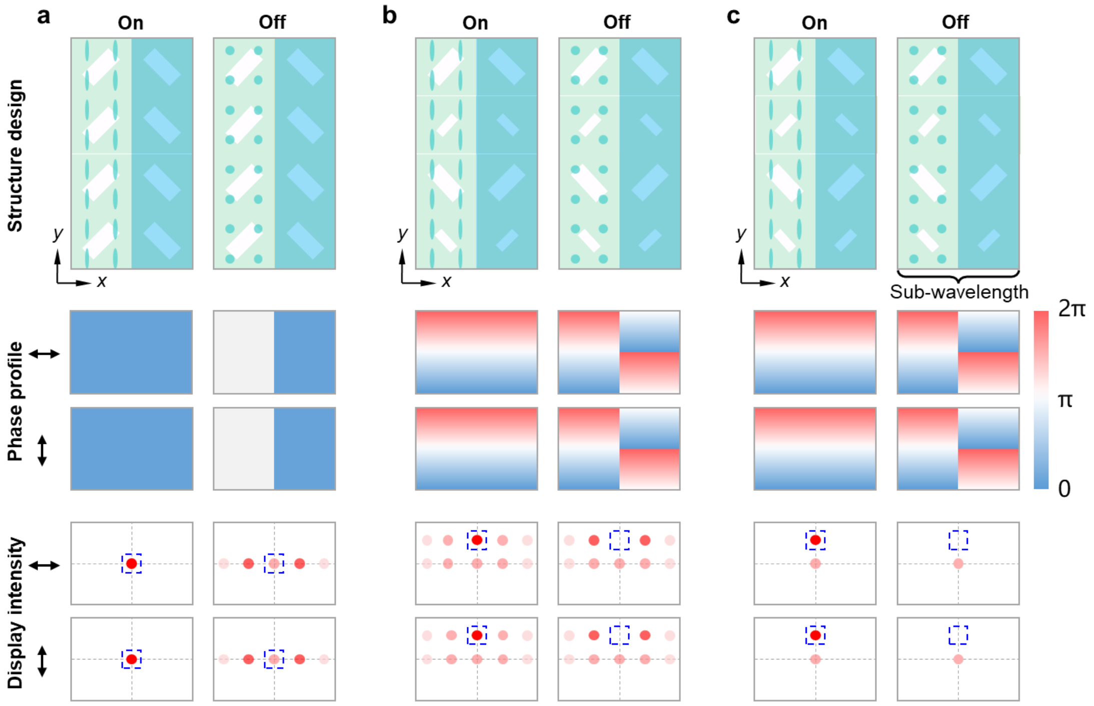

Supplementary Fig. 3. Schematic of the three designed metasurface super unit cells, along with the corresponding phase profiles and resulting display intensities. a, The methodology for realizing optical switch based on phase difference in the *x*-direction. **b,** Add a phase gradient in the *y*-direction to method **(a)** to achieve higher contrast off-axis reflection points. **c,** Reduce the period in the **x**-direction in method **(b)** to avoid higher-order diffraction. The black arrows indicate the polarization states of the incident light. The blue dashed box indicates the designed switching point. In the main text, we employ the design methodology shown in **c**.

Section 4: Numerical simulation of metasurface unit cell

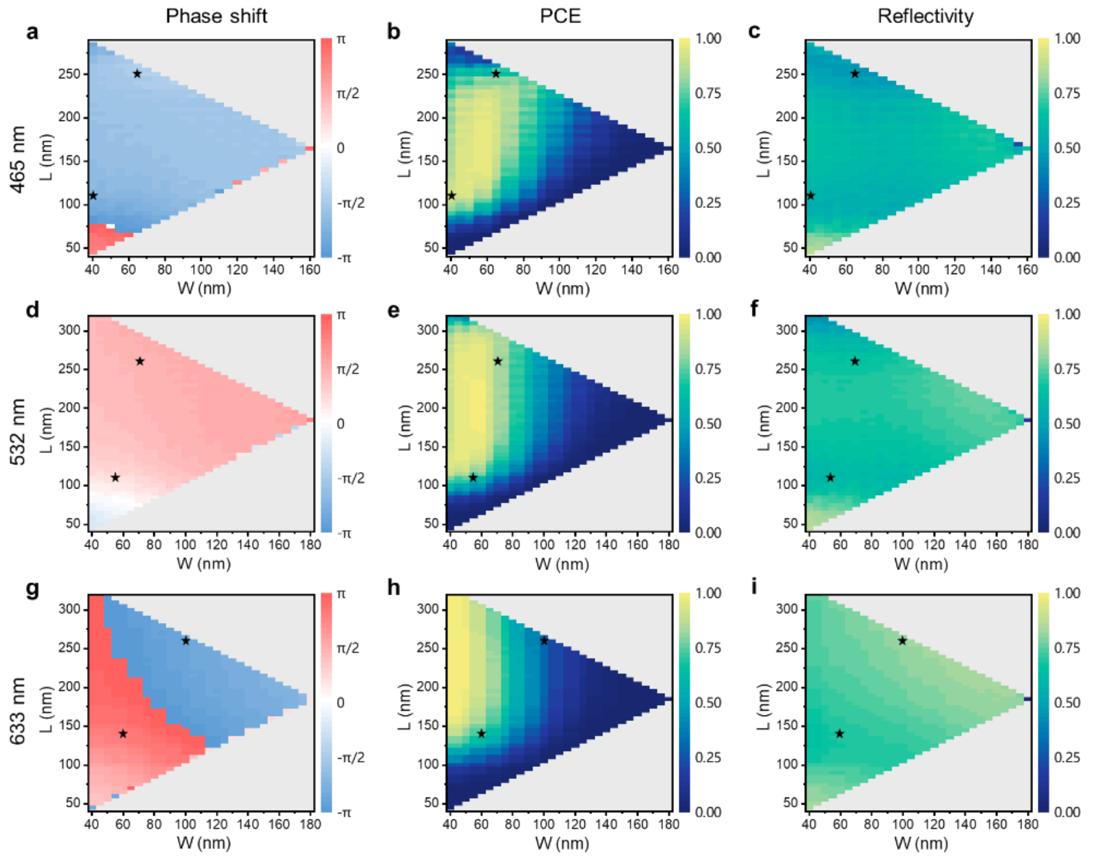

Supplementary Fig. 4. Numerical simulation results of the unit cell at operational wavelengths of 465 nm, 532 nm and 633 nm, respectively. Phase shift (**a/d/g**), polarization conversion efficiency (**b/e/h**) and reflectivity (**c/f/i**) of nanorods of varying sizes at operational wavelength of 465/532/633 nm. The stars indicate the two nanorods selected to construct the metasurface super unit cell.

Each super unit cell contains four levels of phase delays of 0, π/2, π and 3π/2 in sequence, so two types of cells with a phase difference $i\pi/2$ (i=1, 3, 5, 7, ⋯) need to be selected and arranged to form the super unit cell, as illustrated in Fig. 2b. The composition of the metasurface unit cell, which is designed as a metal-insulator-metal (MIM) structure of Al-SiO_2-Al, as illustrated in Fig 2c. Critical cell parameters,

including the length (L), width (W), thickness (H) and period (P) of the nanorods as well as the thickness of the SiO_2 (h_1) and spacer layer (h_2), need to be optimized to achieve proper phase, higher polarization conversion efficiency and reflectivity.

The responses of unit cell were simulated by the finite-difference time-domain (FDTD, Ansys Lumerical FDTD) method. For the simulation, the boundary conditions of the x-direction and y-direction were set to periodic boundary conditions. Perfectly matched layers were placed at the top of the simulation area. The refractive index of the SiO_2 and PMMA was taken as 1.5 and the refractive index of the HSQ was taken as 1.4. The refractive index of Si and Al was taken from Palik in the FDTD software. The rotation angle of the nanorods was pre-set to 45°. Constraints ($L > W$ and $L+W < \sqrt{2}P$) were added to void nanorods out of period and improve the efficiency of the simulation. Considering the limitations of experimental conditions, we set the minimum size constraints of the nanorods to be 40 nm with a variation of 5 nm. To simplify the fabrication process, the thickness of each layer at each of the three operating wavelengths (465 nm, 532 nm, and 633 nm) was defined as the same parameter. By sweeping and optimizing the parameters, the final thickness of each layer was determined as follows: 200 nm for the aluminum mirror, h_1 = 50 nm for the SiO_2, h_2 = 70 nm for the spacer layer and H = 50 nm for the nanorods. Phase shift, polarization conversion efficiency and reflectivity for the x-polarization incident light as a function of the length and width of the nanorods as shown in Supplementary Fig. 4. The stars indicate the two nanorods selected to construct the metasurface. Two

types of nanorods (P = 260 nm) with dimension of 110 nm × 55 nm and 260 nm × 70 nm at 532nm (250 nm × 65 nm and 110 nm × 40 nm at 465 nm with P = 230 nm, as well as 260 nm × 100 nm and 140 nm × 60 nm at 633 nm with P = 260 nm) are selected and arranged to form the super unit cell. The spectral response of the selected unit cells also be simulated, as shown in Supplementary Fig. 5.

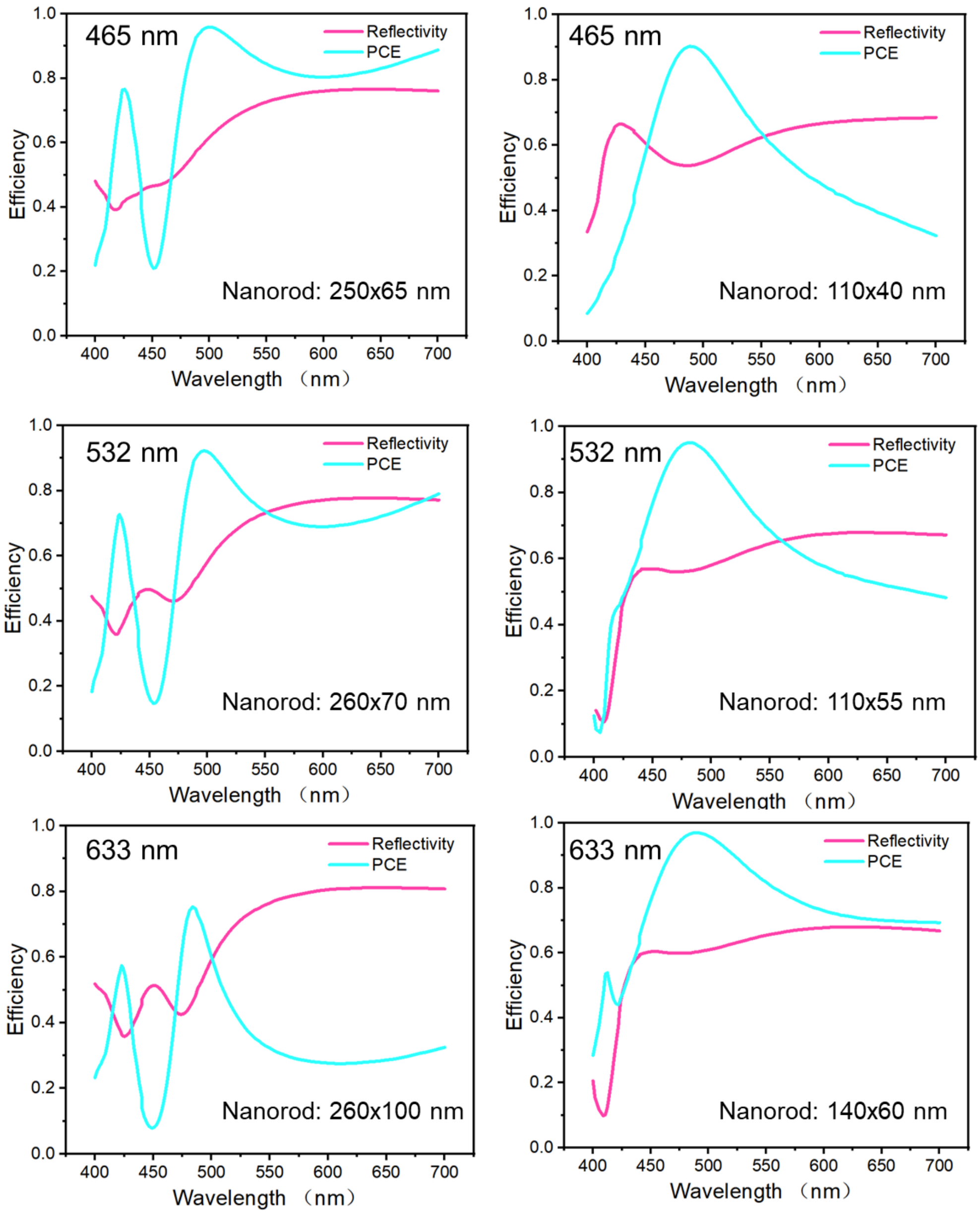

Supplementary Fig. 5 The simulated spectral response of the selected unit cells at the three operational wavelengths.

Section 5: The intrinsic parameters of the high-birefringence LC

Supplementary Tbl. 1 The intrinsic parameters of high-birefringence LC

Clearing Point (°C)	124.4
Rotary Viscosity (mpa.s, 70°C)	100.6
Δn (532 nm, 25°C)	0.42
n_o (532 nm, 25°C)	1.5
$\Delta\varepsilon$ (1KHz, 70°C)	15.6
$\varepsilon_{\perp}$(1KHz, 70°C)	4.2
K_{11}(pN, 25°C)	20.1
K_{22}(pN, 25°C)	/
K_{33}(pN, 25°C)	25.6

Section 6: The effect of fabrication errors on the performance of device

1. Overlay error

To quantitatively assess the impact of overlay errors on device performance, we have simulated the total efficiency of the device (the total efficiency is equal to the product of the diffraction efficiency and reflectivity) across a range of overlay errors (0-100 nm). As illustrated in Supplementary Fig. 6, the efficiency decreases gradually with the increase of overlay errors and degrades particularly sharply beyond the 90 nm. This abrupt transition indicates that the device is beginning to fail. Consequently, we have set 90 nm as the upper tolerance limit for overlay accuracy in the fabrication process.

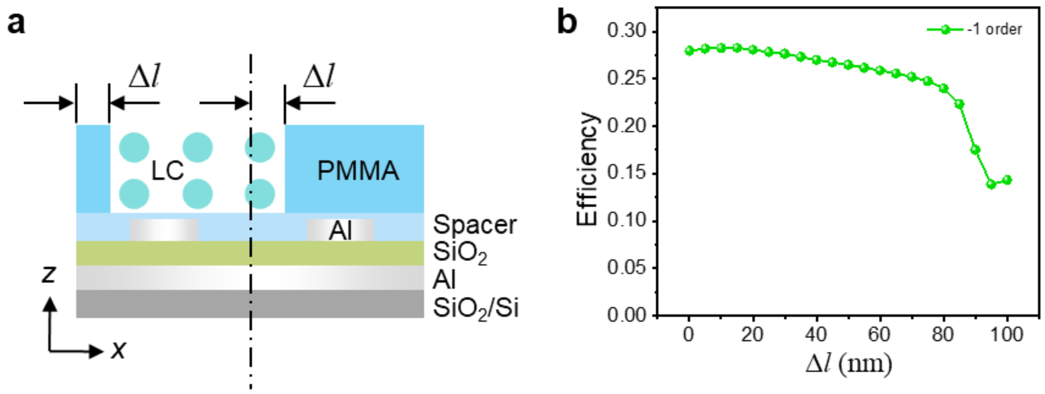

Supplementary Fig. 6. The effect of overlay errors on device efficiency. a, Schematic diagram of overlayer error in a supercell. **b,** Simulated total efficiency of the off-axis reflection light at different overlayer error with operational wavelength of 532 nm.

2. Dimensional errors of nanorods

As illustrated in Supplementary Fig. 7, the device maintains stable performance when dimensional errors of nanorods remain within -20 nm of the designed dimensions. When dimensional errors exceed +5 nm, we observe a particularly sharp decline in device efficiency. This behavior can be attributed to the resonant nature of the metasurface elements. Within the -20 nm tolerance range, the nanorods remain within their designed resonant regime, preserving both the polarization conversion efficiency and phase modulation accuracy. However, when nanorods dimensions deviate beyond this range, the meta-atoms shift out of their optimal resonance condition, leading to degraded polarization conversion and disrupted phase gradient profile, which collectively account for the observed efficiency reduction. These findings establish clear fabrication tolerance limits while demonstrating the robustness of our design to minor process variations characteristic of high-volume semiconductor

manufacturing.

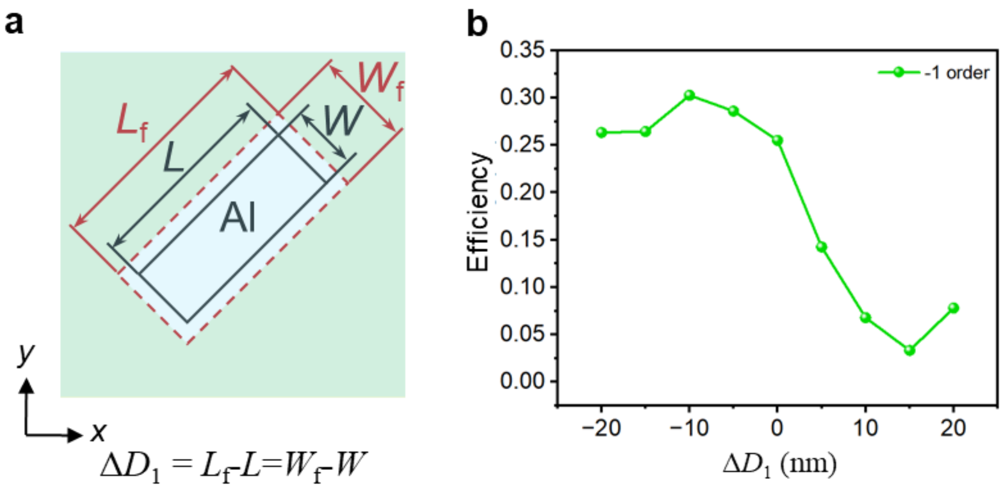

Supplementary Fig. 7 The effect of dimensional errors of nanorods on device efficiency. a, Schematic diagram of dimensional errors of nanorods. L and W are designed dimensions, while L_f and W_f are actual fabrication dimensions. **b,** Simulated total efficiency of the off-axis reflection light at different dimensions error with operational wavelength of 532 nm.

3. Dimensional error of the nanograting

To evaluate the fabrication tolerance of nanograting, we conducted numerical simulations to analyze the relationship between grating dimensional variations and optical efficiency. As illustrated in Supplementary Fig. 8, the device maintains stable performance across a wide range of grating size errors (±40 nm), with efficiency fluctuations remaining within small value of the nominal value. This remarkable stability suggests that our design exhibits substantial robustness against typical fabrication variations encountered in nanoscale manufacturing processes.

The observed insensitivity to dimensional variations can be attributed to the non-resonant operation principle of the grating, where slight deviations from ideal

dimensions do not significantly alter the phase modulation characteristics. These simulation results confirm that our device architecture can accommodate the inherent process variations of standard semiconductor fabrication techniques while maintaining consistent optical performance, a critical requirement for practical implementation and mass production.

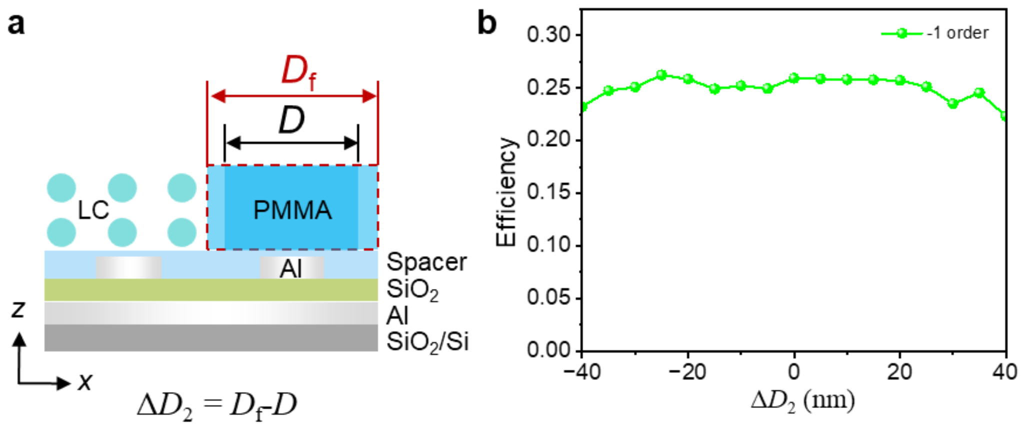

Supplementary Fig. 8 The effect of dimensional errors of nanograting on device efficiency. a, Schematic diagram of dimensional errors of nanograting. *D* is designed dimension and D_f is actual fabrication dimension. **b,** Simulated total efficiency of the off-axis reflection light at different dimensions error with operational wavelength of 532 nm.

Section 7: Discussion on the decline in experimental contrast

The experimentally measured contrast ratios at operational wavelengths exhibit deviations from simulated predictions, primarily attributable to refractive index discrepancies of PMMA nanogratings and fabrication-induced structural imperfections.

The first reason for this discrepancy is that the refractive index of the

manufactured PMMA nanograting differ from the design values. In contrast, the refractive index of the two materials is set to be equal in the simulation. This mismatch in the experimental device results in incomplete destructive interference in the “off” state. The refractive index of PMMA ranges between 1.49 and 1.51 across the visible region[1]. However, the fabricated PMMA nanograting exhibits a reduced effective refractive index (approximately 1.48 at λ = 532 nm, as quantified through spectroscopic ellipsometry in Supplementary Fig. 9a) due to porosity-induced structural variations.

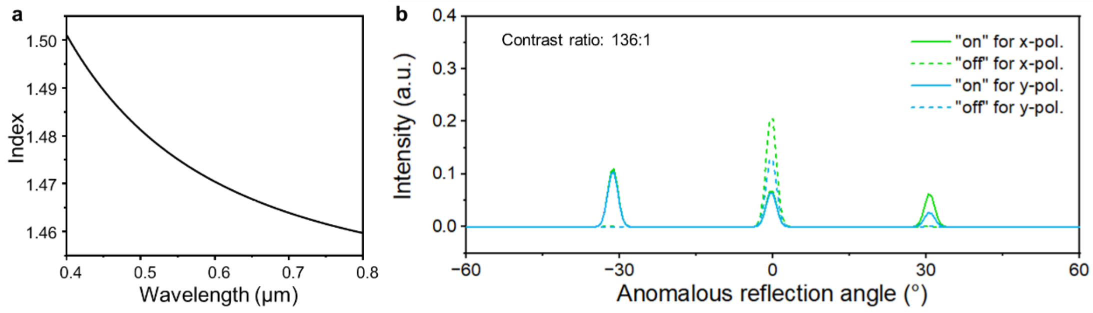

Supplementary Fig. 9 a, The refractive index of PMMA in the experiment. **b,** Numerical contrast ratio with the PMMA refractive index n_G = 1.48 at the operating wavelength of 532 nm.

To quantitatively assess the impact of refractive index variations on device contrast, we have performed numerical simulations with a nanograting refractive index of n_G = 1.48. As shown in Supplementary Fig. 9b, this configuration yields a simulated contrast ratio of 136:1, which is slightly higher than the contrast ratio achieved in the experiment.

Furthermore, fabrication and packaging imperfections contribute significantly to contrast ratio degradation. Structural deviations—including dimensional inaccuracies,

morphological defects, and sidewall steepness variations—compromise the designed phase gradient fidelity, thereby attenuating destructive interference in the “off” state. During encapsulation, incomplete LC infiltration induces microbubble formation, further distorting the phase gradient of the device. These combined effects reduce the maximum achievable contrast compared to ideal simulated conditions.

To mitigate these effects and improve the contrast ratio, we propose the following solutions. To address the refractive index mismatch between PMMA and the n_o of the LC, strategic doping of high-index nanoparticles (e.g., TiO_2, n≈2.5 at 532 nm) into the PMMA presents a viable solution[2, 3]. The use of another nanograting materials with refractive index that more closely match the n_o of the LC could also enhance the contrast ratio. An alternative approach involves enhancing the fabrication process to achieve higher precision in structures production. By implementing advanced lithographic techniques and optimized deposition methods, we can minimize structural imperfections that lead to the designed phase gradient fidelity variations. This includes reducing material porosity and other fabrication-induced defects.

Section 8: Switching rate characterization

Supplementary Fig. 10 illustrates the switching time of the polarization-insensitive meta-LCoS device operating at a wavelength of 532 nm. The device is switched between “on” and “off” states by applying voltages of 0 V and 30 V, respectively. The time required to switch the modulation intensity from 90% to 10%

of the switching window is defined as the fall time and rise time, respectively. The intensity of the off-axis reflection light generated by the meta-LCoS device was quantified using an optical power meter with a sampling interval of 4 ms, yielding final fall time and rise time were measured to be 40 ms and 56 ms, respectively. These times are primarily influenced by the intrinsic properties of the LC, the thickness of the LC layer, and the anchoring force of the orientation layer.

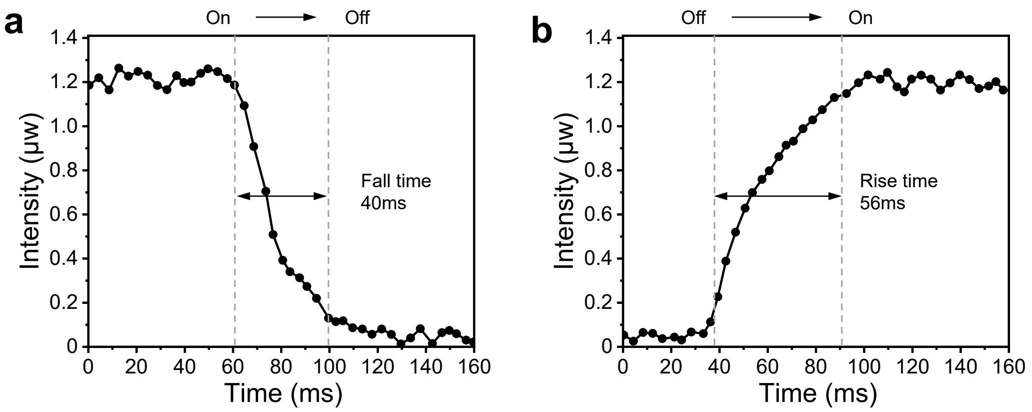

Supplementary Fig. 10. Switching time characterization of polarization-insensitive meta-LCoS device at operational wavelengths of 532 nm. a, The switching time from “on” to “off” states. **b,** The switching time from “off” to “on” states.

Section 9: Fabrication of multi-pixel Al electrodes

The preparation of the multi-pixel samples began with the fabrication of Al pixel electrodes as a preliminary step. The fabrication process for the Al pixel electrodes is illustrated in Supplementary Fig. 11. First, a 500 nm photoresist was spin-coated onto a 30 × 30 mm silicon substrate with a 285 nm oxide layer. Electrode patterns and alignment marks were defined on the substrate using laser direct writing (LDW) lithography. Next, a 5 nm Cr adhesion layer and a 200 nm Al film were deposited via

thermal evaporation followed by a lift-off procedure to create Al pixels and alignment marks. Subsequently, a 50 nm SiO_2 spacer layer was meticulously fabricated on the Al electrodes using ion beam sputtering. The remaining steps are identical to those used for creating single-pixel samples, except that the Al electrode pads must be carefully protected throughout the fabrication process. The fabricated 64-pixel Al electrode array is shown in Supplementary Fig. 12.

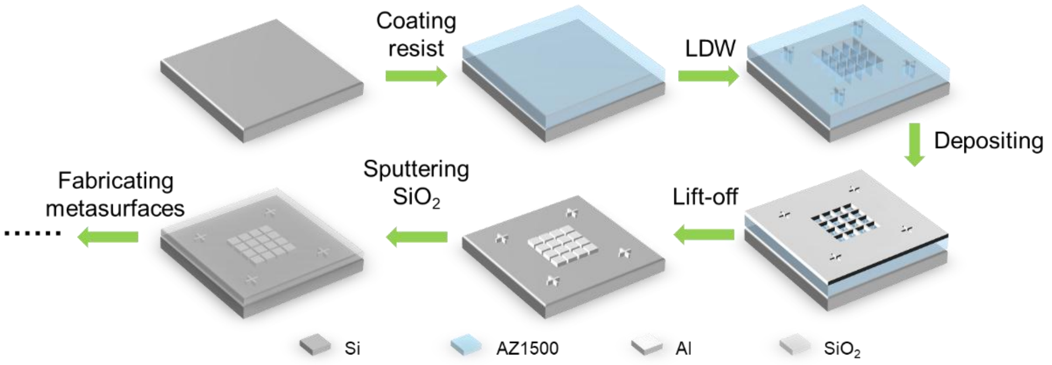

Supplementary Fig. 11. Fabrication process of Al pixel electrodes. Once the Al pixel electrodes were fabricated, the metasurface was fabricated on the electrodes using the EBL overlay technique.

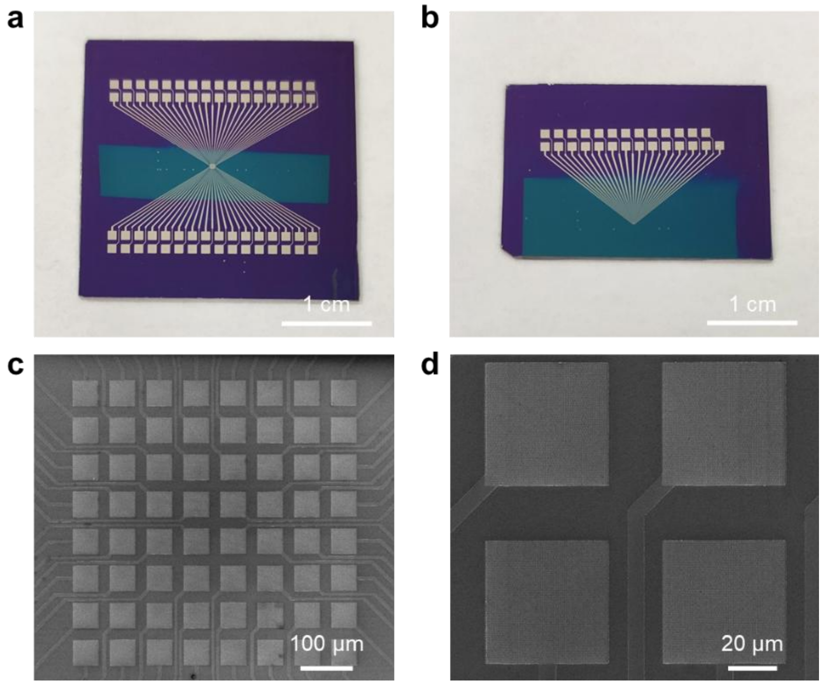

Supplementary Fig. 12. Fabricated multi-pixel Al electrode arrays. a, Photograph of the

fabricated 64-pixel Al electrode array. The light blue area is sputtered with a 50 nm layer of SiO_2. Scale bar is 1 cm. **b,** Photograph of the fabricated 9-pixel Al electrode array containing 27 subpixels for the monolithic color meta-LCoS display. The light blue area is sputtered with a 50 nm layer of SiO_2. Scale bar is 1 cm. **c,** SEM image of the metasurface fabricated onto the 64 Al pixels using the EBL overlay technique. Scale bar is 100 μm. **d**, Magnified SEM image of the fabricated metasurface. Scale bar is 20 μm.

Section 10: Testing setup for polarization-insensitive meta-LCoS device

Supplementary Fig. 13 illustrates the photograph of the testing setup for polarization-insensitive meta-LCoS prototype device. The unpolarized light emitted by the LED light source is collimated by a 4f system consisting of two lenses, ensuring direct illumination of the designed meta-LCoS. An iris is used to regulate the dimensions of the light spot projected onto the meta-LCoS. The pattern generated by the meta-LCoS is transmitted through the projection lens and captured by a CCD camera. The CCD camera can be replaced by a photodetector to quantify the intensity of the off-axis reflection light. The digital drive signals for the meta-LCoS are generated by a 64-channel digital controller developed in-house, which can receive input from a computer. The polarization-insensitive meta-LCoS in this work has the potential to significantly streamline conventional LCoS projection systems by eliminating the need for intricate polarization components within the test setup. This simplified design markedly reduces system complexity and, consequently, production costs.

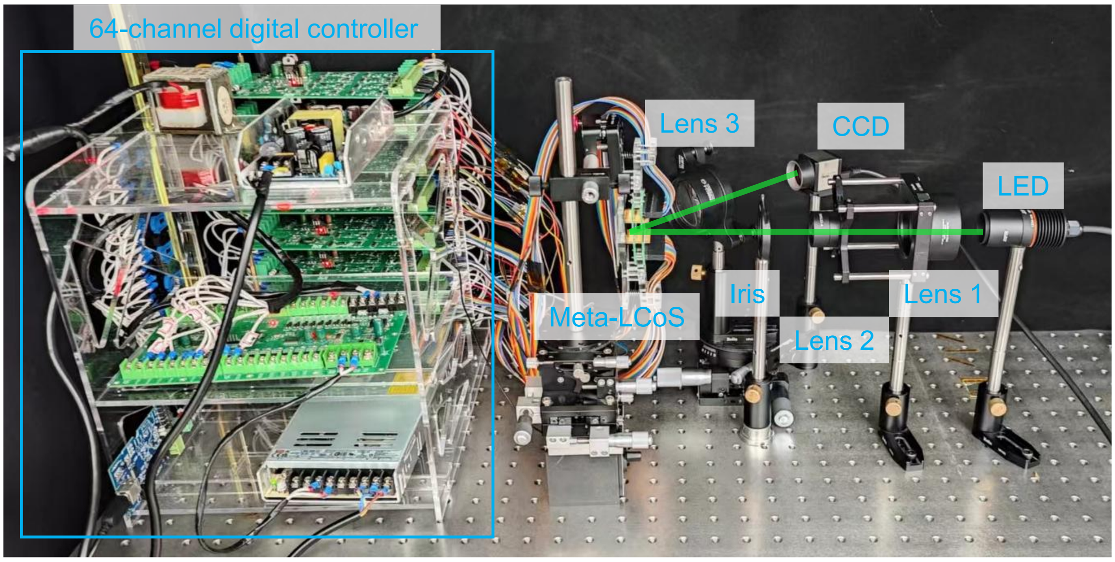

Supplementary Fig. 13. Photograph of the testing setup for polarization-insensitive meta-LCoS device.

Section 11: Meta-LCoS optical engine for color projection display

We have considered an optical engine for color projection display based on the designed polarization-insensitive monochrome meta-LCoS with operating wavelengths of 465 nm (blue), 532 nm (green), and 633 nm (red), respectively, as illustrated in Supplementary Fig. 14. The light source is first spectrally separated into its constituent red, blue, and green light beams by a beam-splitting dichroic prism. These monochromatic beams are then sequentially projected onto three distinct meta-LCoS panels. The individual images, each rendered in a primary color, are subsequently integrated to synthesize a full-color image. It is imperative to highlight that the proposed design serves as an example for color projection display. Although this configuration demonstrates the fundamental principles, it is possible that alternative setups may be required to meet the specific requirements of each use case,

ensuring the optimal and most efficient color projection optical engine architecture is achieved.

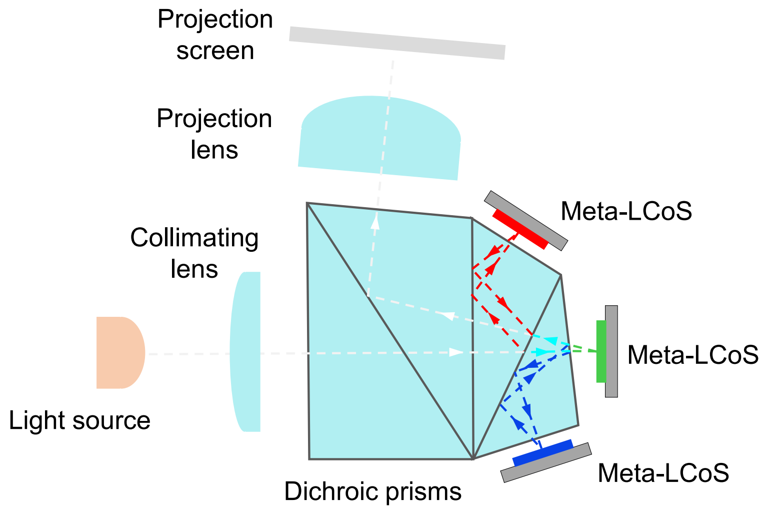

Supplementary Fig. 14. Polarization-insensitive meta-LCoS optical engine for color projection display.

Section 12: Polarization-insensitive LCoS device realized with dielectric nanopillars

An alternative approach we have explored involves integrating narrowband color filters with the metasurface subpixels, as shown in Supplementary Fig. 15. In this configuration, the narrowband filters are aligned with the subpixels of the metasurface to separate the incident broadband light while the nanorod periods are similarly optimized to maintain off-axis angle consistency for all wavelengths. This filtered approach offers potential advantages in pixel crosstalk mitigation, drawing upon established applications of spectral filtering in high-resolution display technologies. Both methods mentioned in our work demonstrate viable pathways toward achieving

full-color operation in monolithic meta-LCoS devices, with the choice between them depending on specific performance requirements and implementation constraints.

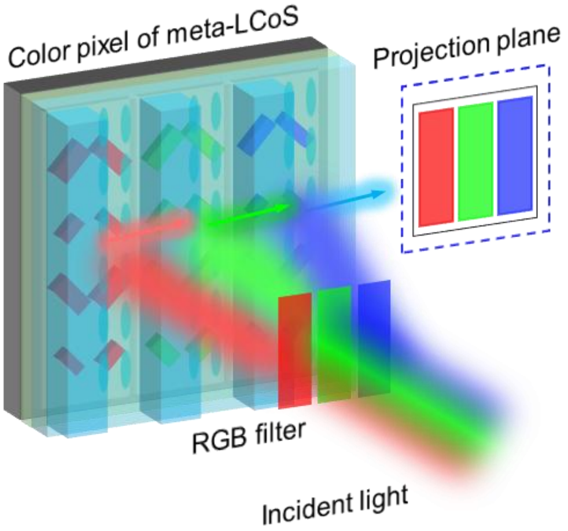

Supplementary Fig. 15. Schematic of the monolithic color meta-LCoS projection display implemented by utilizing color filter.

Section 13: Polarization-insensitive LCoS device realized with dielectric nanopillars

In the visible region, dielectric materials are optimal for the realization of highly efficient metasurface devices due to their reduced loss and absorption characteristics. In this study, we considered the use of dielectric nanopillar structures in place of aluminum nanorods to achieve the design methodology illustrated in Supplementary Fig. 3c. The propagation phase and geometric phase of the dielectric nanopillars are employed in the design of the off-axis reflection light generation. As previously, the phase shift, polarization conversion efficiency, and reflectivity of the unit cell were numerically simulated. It is regrettable that the polarization conversion efficiencies of

the dielectric nanopillars are typically insufficient due to the limited lattice period in our numerical simulations, which hinders the selection of suitable nanopillars for constructing the super unit cell in Supplementary Fig. 3c.

Ultimately, we adopted the methodology shown in Supplementary Fig. 3b for constructing dielectric nanopillars. The optimized dielectric nanopillars arrangement of the metasurface super unit cell, as shown in Supplementary Fig. 16a-c, was obtained through numerical simulations at the operational wavelengths of 465 nm, 532 nm, and 633 nm, respectively. The specific geometric parameters of dielectric nanopillars are shown in Supplementary Tbl. 2. The boundary conditions of the *x*-direction and *y*-direction were set to periodic boundary conditions. Perfectly matched layers were placed at the top of the simulation area. To maximize the conversion efficiency, the metasurface design has been optimized by increasing the density of nanopillars at the operational wavelength of 465 nm. For the operational wavelengths of 532 nm and 633 nm, the nanopillars were strategically designed to extend beyond the conventional unit cell period. It is important to note that, in the FDTD numerical simulations, the regions of the nanopillars that extend beyond the unit cell period were not included. The exclusion has a diminished impact on the optical properties of the entire super unit cell, as the core interaction region between light and the nanopillars are concentrated inside the unit cell. Finally, intensity modulation with contrast ratios of 4390:1, 3817:1 and 2627:1, along with light utilization values of 0.57, 0.55 and 0.56, was achieved at 465 nm, 532 nm and 633 nm wavelengths, respectively. Light

utilization is defined as the ratio of the intensity of off-axis reflection light in the “on” state to the intensity of the incident light.

However, the fabrication of this super unit cell necessitates stringent accuracy in the manufacturing process. Minor deviations in the fabrication process can lead to alter the designed phase gradient, potentially resulting in reduced efficiency or even the failure of the metasurface to achieve its intended optical effects. Supplementary Fig. 17 shows the far-field simulation results from dielectric polarization-insensitive LCoS at the operational wavelength of 532 nm. Since subwavelength modulation is not feasible, the far-field diffraction pattern exhibits a significant presence of higher order diffraction levels. The higher-order diffracted light will directly result in a degradation of the image quality of the projection display. Given the inherent difficulties in fabricating dielectric nanopillars with the requisite precision in lab, we ultimately opted for the aluminum structure outlined in the main text to fabricate the device.

Further optimizing the lattice design of the metasurface can improve the feasibility of dielectric nanopillars. Multi-objective inverse design can be achieved using non-local design or intelligent algorithms that adjust parameters such as the period, shape, and size of the nanopillars. This facilitates the creation of metasurface lattices with excellent optical performance and low manufacturing difficulty. Additionally, the dielectric system can be used as an optional solution for subsequent mass production with the advanced CMOS process. Although we chose TiO_2 in our

simulation, materials with better process compatibility, such as Si_3N_4 and SiC, can also be selected. These dielectric materials offer improved CMOS compatibility while maintaining suitable optical properties. The specific preparation process can also be further optimized. Advanced lithography solutions including deep ultraviolet lithography and nanoimprint lithography can replace electron beam lithography to achieve high-resolution, low-cost patterning at production scales. When combined with coating and etching processes, these solutions enable stable, high-yield structural processing. Planarization techniques leveraging chemical-mechanical polishing with optimized slurries ensure surface uniformity for subsequent processing steps. Finally, the preparation of nanogratings is achieved by photolithography overlay processes. Therefore, the dielectric nanopillars scheme shows great potential for mass production and commercial viability.

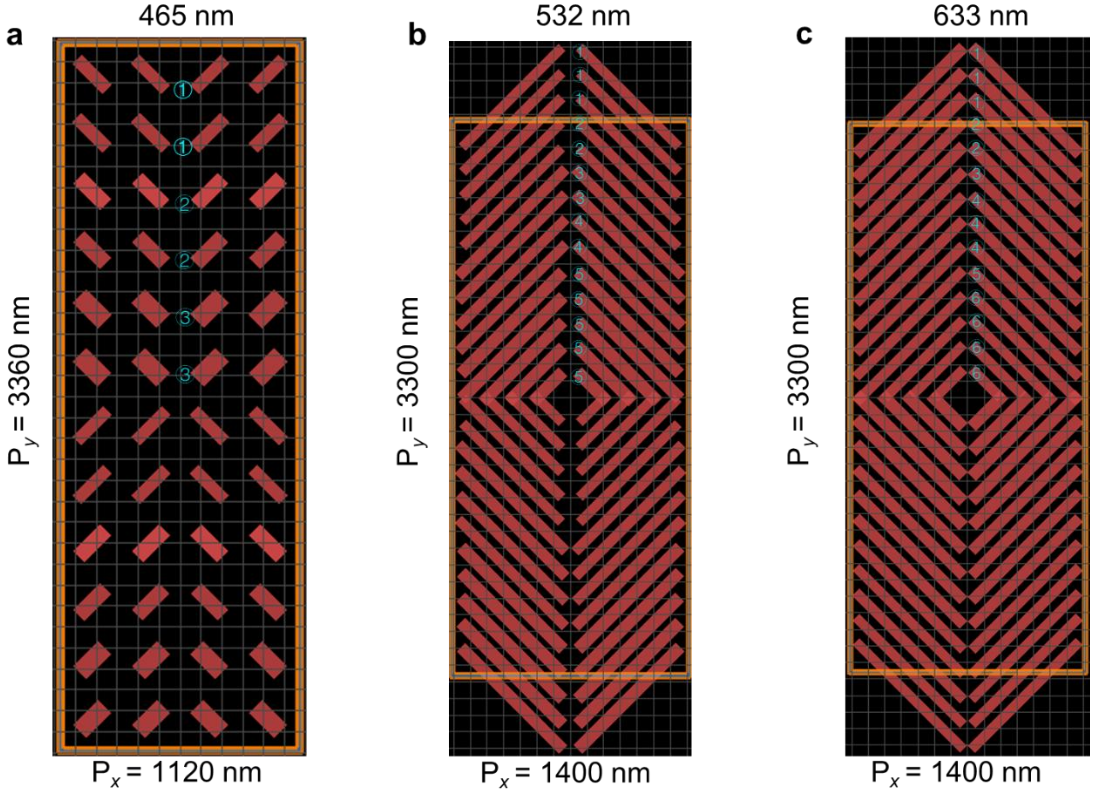

Supplementary Fig. 16. Dielectric nanopillars arrangement of the metasurface super unit cell after optimization in numerical simulations at the operational wavelength of 465 nm (a), 532 nm (b), and 633 nm (c), respectively. The orange rectangular box represents the boundary of the simulation cycle, the red rectangle represents the nanopillars, and the gray thin lines are the measurement grid with 100 nm × 100 nm. The geometric parameters of the nanopillars and SiO_2 layer are optimized as presented Supplementary Tbl. 2.

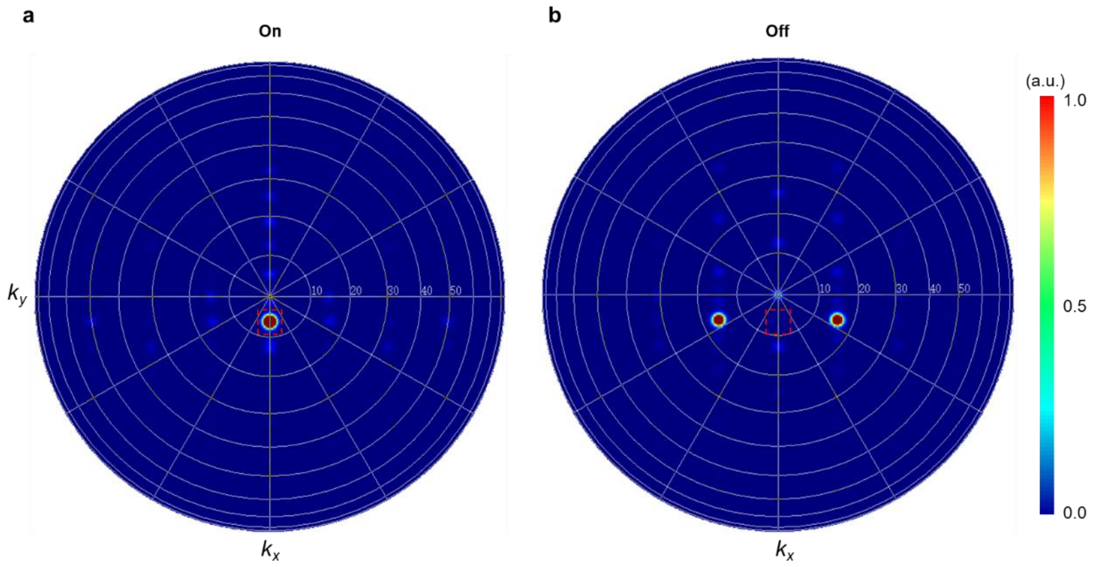

Supplementary Fig. 17. Far-field simulation results from dielectric polarization-insensitive LCoS at the operational wavelength of 532 nm. a, The "on" state of optical switch. **b**, The "off" state of optical switch. Since subwavelength modulation is not feasible, the far-field diffraction pattern exhibits a significant presence of higher order diffraction levels.

Supplementary Tbl. 2. Geometric parameters of dielectric nanopillars at the operational wavelength of 465 nm, 532 nm, and 633 nm, respectively. A slight adjustment to the dimensions of the structure results in a smoother phase gradient.

Parameter \ Wavelength	465 nm	532 nm	633 nm
Height of nanopillars (nm)	700		
Height of spcer layer (nm)	705		
Height of SiO_2 (nm)	200	120	150
Nanopillars 1 (nm)	206×60	844.44×60	920×73
Nanopillars 2 (nm)	184×75	840×60	920×74.5
Nanopillars 3 (nm)	170×90	911.11×65.5	920×66.5
Nanopillars 4 (nm)	/	911.11×64.44	920×65
Nanopillars 5 (nm)	/	866.67×74	920×62
Nanopillars 6 (nm)	/	/	920×60
Period of nanopillars (nm)	280×280	700×150	

Section 14: Parameter comparison of different microdisplay technologies

Supplementary Tbl. 3. Parameter comparison of different microdisplay technologies.

**Parameters**	**DMD**	**Conventional LCoS**	**Our meta-LCoS**
Lighting source	unpolarized illumination	polarized illumination	unpolarized illumination
Maximum resolution	4K	8K and beyond	8K and beyond
Contrast ratio	~10^3:1	~10^3:1	~10^3:1[1]
Color display	three-chip or time-multiplexed monolithic	three-chip or time-multiplexed monolithic	Non-time multiplexed monolithic
Smallest pixel pitch	>5 μm	>3 μm	<3 μm[2]

[1]: data from simulation; [2]: theoretical data.

Supplementary References

1. Beadie, Guy, et al. Refractive index measurements of poly (methyl methacrylate) (PMMA) from 0.4–1.6 μm. *Applied optics* **54**, 31: F139-F143 (2015).
2. Chatterjee, Amit. Properties improvement of PMMA using nano TiO_2. *Journal of applied polymer science* **118**, 5: 2890-2897 (2010).
3. Sugumaran, S., and C. S. Bellan. Transparent nano composite PVA–TiO_2 and PMMA–TiO_2 thin films: Optical and dielectric properties. *Optik* **125**, 18: 5128-5133 (2014).
