# Supplementary material for: Meta-optics redefines microdisplay: monolithic color LCoS without polarization dependency
Source: Nat Commun. 2025 Dec 8;16:10925. doi: 10.1038/s41467-025-66032-z (PMC12685945; doi:10.1038/s41467-025-66032-z)
Supplement: Supplementary file 2 — Description of Additional Supplementary Files [file 41467_2025_66032_MOESM2_ESM.pdf]

Description of Additional Supplementary Files:

Supplementary Movie 1: Optical sw itch at 465 nm.

Supplementary Movie 2: Optical sw itch at 532 nm.

Supplementary Movie 3: Optical sw itch at 633 nm.

Supplementary Movie 4: Dynamic projection display of the three letters “h”, “n” and “u”.

Supplementary Movie 5: Dynamic projection display of numbers “0-9”.

Supplementary Movie 6: Dynamic projection display of upw ard sign formed by arrows.

Supplementary Movie 7: Dynamic projection displays at operational w avelength of 465 nm.

Supplementary Movie 8: Dynamic projection displays at operational w avelength of 532 nm.

Supplementary Movie 9: Dynamic projection displays at operational w avelength of 633 nm.

Supplementary Movie 10: Projection display of color pixels in the monolithic color meta-LCoS.
